# Global Transcriptomic Profiling of Cardiac Hypertrophy and Fatty Heart Induced by Long-Term High-Energy Diet in Bama Miniature Pigs

**DOI:** 10.1371/journal.pone.0132420

**Published:** 2015-07-10

**Authors:** Jihan Xia, Yuanyuan Zhang, Leilei Xin, Siyuan Kong, Yaoxing Chen, Shulin Yang, Kui Li

**Affiliations:** 1 State Key Laboratory of Animal Nutrition, Institute of Animal Sciences, Chinese Academy of Agricultural Sciences, Beijing, P. R. China; 2 College of Veterinary Medicine, China Agricultural University, Beijing, P. R. China; 3 Agricultural Genomes Institute at Shenzhen, CAAS, Shenzhen, 518120, P.R. China; University of Catanzaro Magna Graecia, ITALY

## Abstract

A long-term high-energy diet affects human health and leads to obesity and metabolic syndrome in addition to cardiac steatosis and hypertrophy. Ectopic fat accumulation in the heart has been demonstrated to be a risk factor for heart disorders, but the molecular mechanism of heart disease remains largely unknown. Bama miniature pigs were fed a high-fat, high-sucrose diet (HFHSD) for 23 months. These pigs developed symptoms of metabolic syndrome and showed cardiac steatosis and hypertrophy with a greatly increased body weight (2.73-fold, P<0.01), insulin level (4.60-fold, P<0.01), heart weight (1.82-fold, P<0.05) and heart volume (1.60-fold, P<0.05) compared with the control pigs. To understand the molecular mechanisms of cardiac steatosis and hypertrophy, nine pig heart cRNA samples were hybridized to porcine GeneChips. Microarray analyses revealed that 1,022 genes were significantly differentially expressed (P<0.05, ≥1.5-fold change), including 591 up-regulated and 431 down-regulated genes in the HFHSD group relative to the control group. KEGG analysis indicated that the observed heart disorder involved the signal transduction-related MAPK, cytokine, and PPAR signaling pathways, energy metabolism-related fatty acid and oxidative phosphorylation signaling pathways, heart function signaling-related focal adhesion, axon guidance, hypertrophic cardiomyopathy and actin cytoskeleton signaling pathways, inflammation and apoptosis pathways, and others. Quantitative RT-PCR assays identified several important differentially expressed heart-related genes, including STAT3, ACSL4, ATF4, FADD, PPP3CA, CD74, SLA-8, VCL, ACTN2 and FGFR1, which may be targets of further research. This study shows that a long-term, high-energy diet induces obesity, cardiac steatosis, and hypertrophy and provides insights into the molecular mechanisms of hypertrophy and fatty heart to facilitate further research.

## Introduction

Obesity is a worldwide epidemic, especially in industrialized countries, and is associated with heart diseases, including cardiac steatosis, fatty heart and hypertrophy [1]. Western diets rich in both fat and carbohydrates may be responsible for this epidemic [2]. Long-term high energy intake can lead to serious health disorders, including metabolic syndrome, hyperlipidemia, hyperinsulinemia, diabetes, and cardiovascular disease. Obesity has deleterious consequences on heart health and is caused by excessive caloric intake and physical inactivity. The inability to store fat in adipose tissue results in lipid overflow to other organs, such as the liver, pancreas, heart, and skeletal muscle, which usually contain small amounts of fat [3]. In particular, aberrant fat accumulation in the heart has been correlated with the degeneration of the heart muscle surface and the formation of fatty droplets in the sarcolemma [4]. Obese individuals are typically predisposed to increases in heart rate and stroke volume, progressing to ischemic cardiomyopathy, compensatory left ventricular remodeling, nonischemic dilated cardiomyopathy, cardiac fibrosis and apoptosis [5].

The development of fatty heart and hypertrophy are complicated processes, and many hypotheses have been considered with regard to the causal mechanisms. Fat deposits in the pericardium and within the heart initially serve a protective role in energy partitioning [6], but excessive fat deposition has been associated with myocardial damage, inflammation, and heart disease. Higher levels of fatty acids and lipolysis activate the intracellular peroxisome proliferator-activated receptor (PPAR) pathway as well as fatty acid overload, increased oxidation and esterification and triglyceride accumulation [7]. Fatty acid oxidation increases reactive oxygen species (ROS) production, decreases glucose oxidation and insulin sensitivity, and promotes the formation of pro-apoptotic species and intermediates [8]. ROS also activate the mitogen-activated protein kinase (MAPK) pathway, which plays a key role in activating other proteins, mitochondrial dysfunction, heart inflammation and apoptosis [9]. In addition, pressure overload can cause heart hypertrophy through interactions between the actin cytoskeleton, cytokines and focal adhesions [10], progressing to perivascular and myocardial fibrosis and heart failure [11].

Animal models of fatty heart and hypertrophy have been used to characterize lipid storage myopathy and heart dysfunction. However, these disease models have largely involved rodents and are therefore not fully representative of human heart disease. Humans and rodents have obvious metabolic and physiological differences, which have markedly slowed progress and complicated research attempts [12]. Pigs have become a disease model for human metabolic syndrome because of their metabolic similarities to humans, lack of brown fat, and proportional organ sizes and cardiovascular systems [13]. To our knowledge, a pig model of long-term high-energy diet-induced fatty hypertrophy, which would share important similarities with humans, has yet to be reported. This study focuses on the genes and pathways involved in pig heart steatosis and hypertrophy, which may provide meaningful data for further heart research.

## Methods

### Animal and tissue samples

Twelve six-month-old Bama miniature pigs of both sexes were obtained from the Chinese Academy of Agricultural Sciences (CAAS). The miniature pigs used in this experiment were treated humanely according to the “Guide for the Care and Use of Laboratory Animals, ISA, CAAS”, and all procedures were approved by the Animal Care and Use Committee of the Germplasm Resource Center of Chinese Experimental Minipigs. The control group consisted of 6 Bama pigs fed a control diet, and the HFHSD group comprised 6 pigs that were induced with a HFHS diet, which included 37% sucrose, 53% control diet and 10% pork lard. The pigs were separately fed in pens at 30–70% relative humidity and 18–22°C, and they were fed twice every day and provided water *ad libitum* for 23 months. The pigs were fasted for 12 hours and euthanized with ketamine and xylazine. Pig left heart chamber were sampled and preserved in liquid nitrogen.

### Serum test

Pig body weights were measured in addition to the heart weights and volumes. Blood samples were collected after overnight fasting and centrifuged at 3500 rpm for 10 min at 4°C. Serum insulin concentrations were detected using a two-site immunometric assay with monoclonal antibodies. Serum low-density lipoprotein cholesterol, triglycerides and total cholesterol levels were measured with an H7600 autoanalyzer (Hitachi Co., Tokyo, Japan).

### Pig fatty hypertrophic heart

Heart tissues with dimensions of 10 mm×10 mm×2 mm were then submerged in paraformaldehyde, dehydrated in an ascending ethanol series and embedded in paraffin. The heart tissue sections were analyzed by Mallory trichrome staining and hematoxylin and eosin (HE).

### Microarray analysis

Microarray analysis was performed at the Biotechnology Corporation (Shanghai, China). Pig heart total RNA from the HFHSD group pigs (120, 126, 138, 140, 144, and 146) and three control group pigs (157, 159, and 161) were collected using an RNA extraction kit (Qiagen, Valencia, USA), followed by isolation and purification. DNA libraries were constructed according to the preparation manual (Illumina, San Diego, CA, USA). RNA quality was measured with a BioAnalyzer. T7 RNA polymerase-driven RNA synthesis was used to prepare and label cRNAs with Cy3 according to the manufacturer’s instructions. The labeled cRNAs were hybridized to a swine gene microarray according to the manufacturer’s recommendations (Agilent Technologies), and the microarray was then scanned. Agilent Feature Extraction software was used to calculate the raw signal intensities, which were processed using Avadis (Access, Visualize, Analyze, Discover). The raw signal data was normalized and the fold change is the quotient of HFHSD group means divide Control group means scores. Comparative analysis was performed using the Welch t-test with GeneSpring 10.0 to determine the gene expression differences in the HFHSD and control groups. An SBC Analysis System was used to identify the biological processes associated with the differentially expressed genes. The differentially expressed genes were annotated using an online system (SAS system, Shanghai Biotechnology Corporation, China), and gene ontologies (GOs) were determined. The biological pathways associated with the differentially expressed genes were identified using the Kyoto Encyclopedia of Genes and Genomes (KEGG) with Pathway Enrichment Test pvalue<0.05 and qvalue<0.05.

### Real-time QPCR validation

The microarray results were validated by performing real-time QPCR to assess the expression of eleven selected differently expressed genes. Pig heart total RNA was isolated with a TRIzol kit (Invitrogen, Beijing, China) and treated with DNase. The real-time QPCR primers designed are listed in Table 1. The RNA was reverse transcribed to first-strand cDNA using a kit (Promega, Madison, USA). QPCR was performed using a 7500 Real-Time System (Applied Biosystems) with an SYBR Kit (TaKaRa, Dalian, China), following the manufacturer’s protocol. The reaction program was as follows: denaturation at 95°C for 2 min, annealing at 60°C for 30 s, and extension at 72°C for 30 s. The relative expression levels of the genes were calculated from the comparative cycle number (Ct method). Each heart sample was analyzed in triplicate for this experiment.

**Table 1:** Primers used in qRT-PCR for microarray validation.

Gene	Forward primer (5’-3’)	Reverse primer (5’-3’)
STAT3	CCAGGTAGTGCTGCCCCATA	CCTGTCCTCGGCTCCTCA
ACSL4	CCCCACCCACTCCCATCT	GAATTAGCAGCACCCAACCTTA
ATF4	CGCTTCTCACGGCATTCA	GCCCGCCTTAGCCTTGTC
FGFR1	GCGACAGAGGAACAGGGAGG	GCACATTAGTATTGGAATTAAGAAGC
FADD	CGCCTGGACGCCTTCG	TCCTCCCTCCTGCTGTTCTT
PPP3CA	CCCTCCGACGCCAACC	CCCAGAGCAAGTAAGCTACCATC
CD74	CTGAAGCACCTCAAGAACACCA	CAGGTCCTCCGTCTCCAGTG
SLA-8	GCAGTTCGTGCGGTTCG	GGTCCTCGTTCAAAGTGATGTAAT
VCL	TGCTGTAGGGGAAAGTGGC	GGCAGGATATGGGACGGA
ACTN2	CAGCAATGTGCCTGTTCCC	GCACCTGCTTGAAGTAAAATGAA

### Western Blot

The western bolt are performed in three HFSHD group pigs (120, 126, and 146) and three in control group pigs (157, 159, and 161). The HFHSD group and control pigs heart tissue were homogenized and centrifuged with buffer. Protein was performed in Sodium dodecyl sulfate-polyacrylamide gel electrophoresis. The electrophoresis was performed under 140 V in 3 hours, then equilibrating for 15 minutes. The pig heart proteins shifted to nitrocellulose membranes. The membranes were incubated for 1 hour in a blocking buffer. The antibodies against ATF4, Coflin, Hsp27, IKBKG, CD74, ACTN2, PPP3A, CA2 and Actin (Chengdu Zen Bioscience, Chengdu, China) were prepared as diluted 1:500 in buffer (Tris-HCl, pH 7.5, NaCl, Tween-20), then Incubation overnight. The immunoblots were washed in TBS buffer (Tris-Base, NaCl, Tween-20, pH 7.4) and then immersed in the secondary antibody solution to the first antibody containing goat anti-rabbit polyclonal antibodies to horseradish peroxidase. The immunoblotted complexes were detected using an enhanced chemiluminescence Western Blotting Luminal Reagent.

### Statistics

Microarray data analyses were performed using the SAS system (Shanghai Biotechnology Corporation, China). The other statistical data were examined with SPSS 22.0 software. All data were expressed as the mean ± standard deviation (SD), and a P<0.05 was considered statistically significant.

## Results

The HFHSD group pigs displayed obesity, high levels of insulin and dyslipidemia following consumption of the HFHS diet (Table 2) for 23 months. These pigs also showed significant weight gain (P<0.01), increased heart volume (311.67 vs. 195.00, P<0.05), increased heart weight (0.31 vs. 0.17, P<0.05) and a dramatically increased insulin level (4.96-fold; P<0.001) compared with the control pigs, in addition to a 3.91-fold (P<0.01) increase in the fasting serum triglyceride level and a higher low-density lipoprotein cholesterol level (LDL-C) (Table 2).

**Table 2:** Obesity, hyperinsulinemia and dyslipidemia in the Bama miniature pigs.

	HFHSD (n = 6)	Control (n = 6)
Body weight, kg	140.28±8.52**	51.30±5.85
Heart volume, cm^3^	311.67±42.62*	195.00±7.07
Heart weight, kg	0.31±0.04*	0.17±0.00
Insulin, μIU/ml	28.32±7.84**	5.71±0.39
Total cholesterol, mmol/l	3.41±0.29**	1.18±0.38
Plasma triglycerides, mmol/l	1.72±0.32**	0.44±0.19
LDL cholesterol, mmol/l	1.32±0.17*	0.62±0.17

The values are the mean ± standard error.

* P<0.05 and

** P<0.01 compared with the control group.

### Heart fatty deposits and abnormal cardiomyocyte structure and organization in the HFHSD miniature pigs

**Fig 1:**
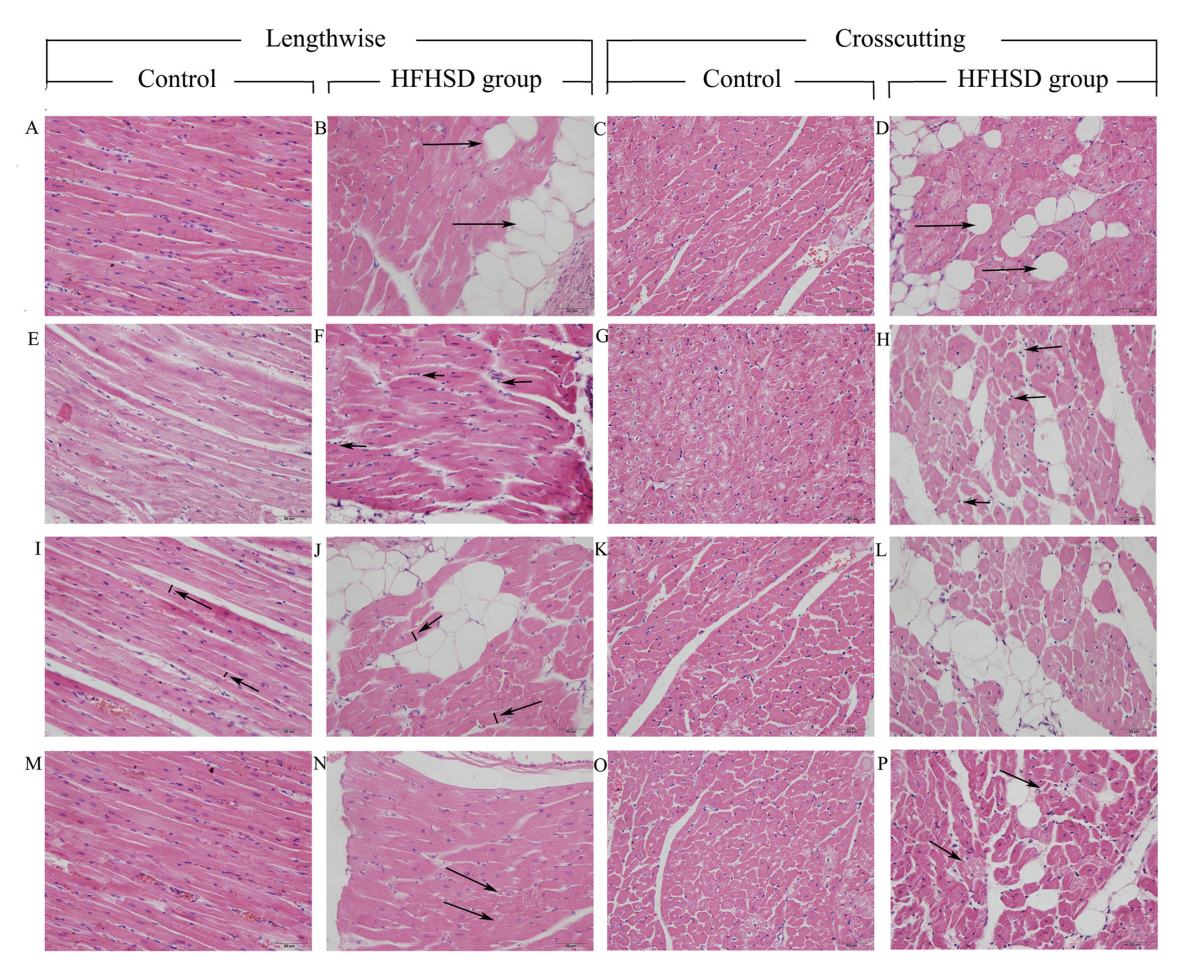
Histopathological changes in miniature pig myocardial tissue (H&E staining). The myocardial fibers from the control miniature pigs were neatly and tightly arranged (A, E, I, M, C, G, K, and O), but they were disorganized in the HFHSD group miniature pigs (arrows in N and P), showing inflammatory cell invasion (arrows in F and H) and cardiomyocyte hypertrophy (arrows in J, L). The tissue of the HFHSD group miniature pigs exhibited fat deposits (arrows in B and D), and some of the myocardial fibers were deformed by fat. A, E, I, M, C, G, K, and O: control group; and B, F, J, N, D, H, L, and P: high-fat and high-sucrose group; bar = 50 μm.

HE staining revealed obvious steatosis in the HFHSD miniature pig hearts compared with those of the control group. The hearts of the HFHSD group miniature pigs were deeply encapsulated by a thick layer of adipose tissue and showed light gray surface coloration along with an increased volume and swelling compared with the control group. When observed under a light microscope, the cardiac myofibers of the pigs in the HFHSD group appeared disorganized, larger, and partially swelled or atrophied. Further, small amounts of inflammatory cells infiltrated the cardiac tissue, and the small blood vessels showed hyperemia. The inflammatory cells are obvious in the cardiac tissue, with relatively large nucleus, darker hyperchromatism, a small amount of cytoplasm. In contrast, cardiac cells show cylindrical cell with the oval or oblong nucleus in the same direction with heart muscle fibers. The interstitial volumes of the myocardial cells of the HFHSD group pigs were enlarged, and large amounts of fat deposits were observed, with some cells being replaced by fat tissue or deformed by the fat. The cardiac myocytes in the control group pigs were normal, compact and aligned, with clearly defined structures (Fig 1).

### Collagenous fibers in the myocardia

**Fig 2:**
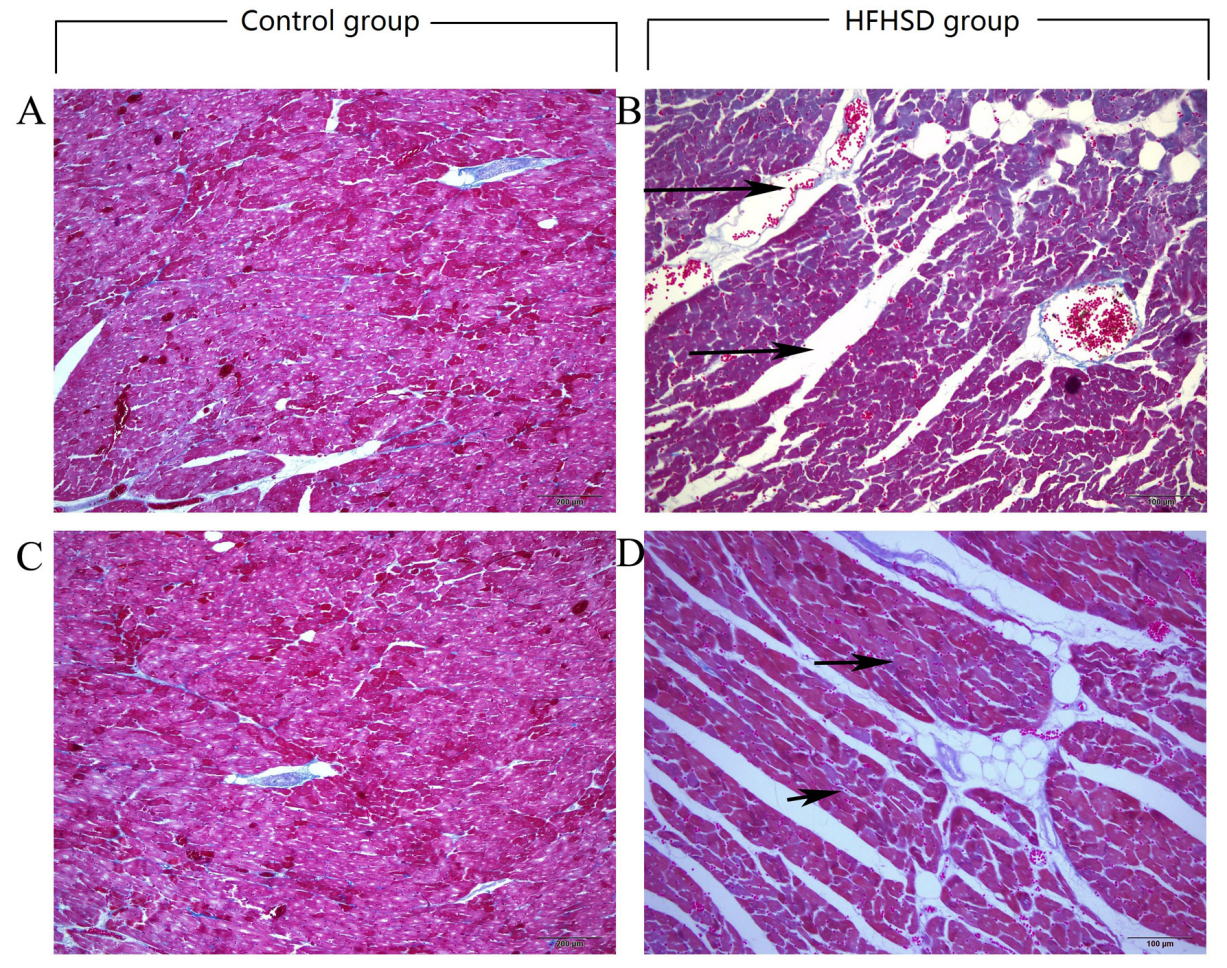
Mallory dye staining showing collagenous myocardial fibers in the two groups. The myocardial fibers were separated by larger distances (arrows in B), and the collagen fibers around the blood vessels were increased (arrows in D) in the HFHSD group. A and C: control group; and B and D: high-fat and high-sucrose group; cross-section, bar = 100 μm.

In the HFHSD group pigs, the heart longitudinal and transverse profiles obtained via Mallory trichrome staining showed larger fat vacuoles deposited in the myocardial fibers, a slight increase in collagen fibers around blood vessels, and thickened small blood vessel basement membranes. The control hearts appeared normal and had clearly defined structures (arrows, Fig 2).

### Gene expression profiles in the HFHSD and control groups

**Fig 3:**
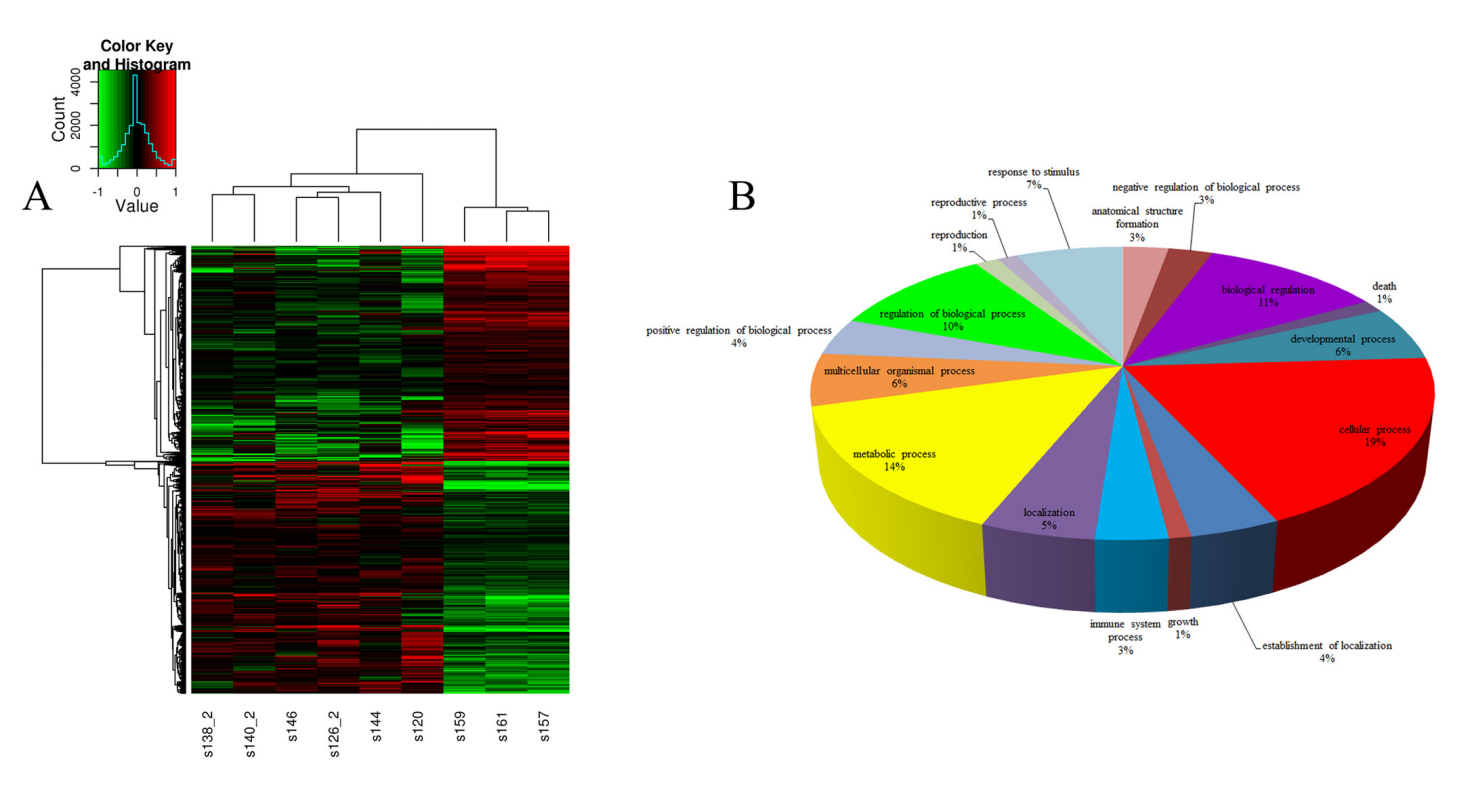
Hierarchical clustering of genes and gene ontology (GO) analysis of biological processes in the HFHSD and control groups. Genes with P values <0.05 were identified as clustered. Red indicates higher expression and green indicates lower expression in the HFHSD group pigs (n = 6) versus the control group pigs (n = 3). Black indicates no difference in expression. The smaller figure depicts the color scale used for clustering (A). Differentially expressed genes between the HFHSD and control groups were used to predict the biological processes involved (B). The GO terms and corresponding percentages are shown in different colors.

**Fig 4:**
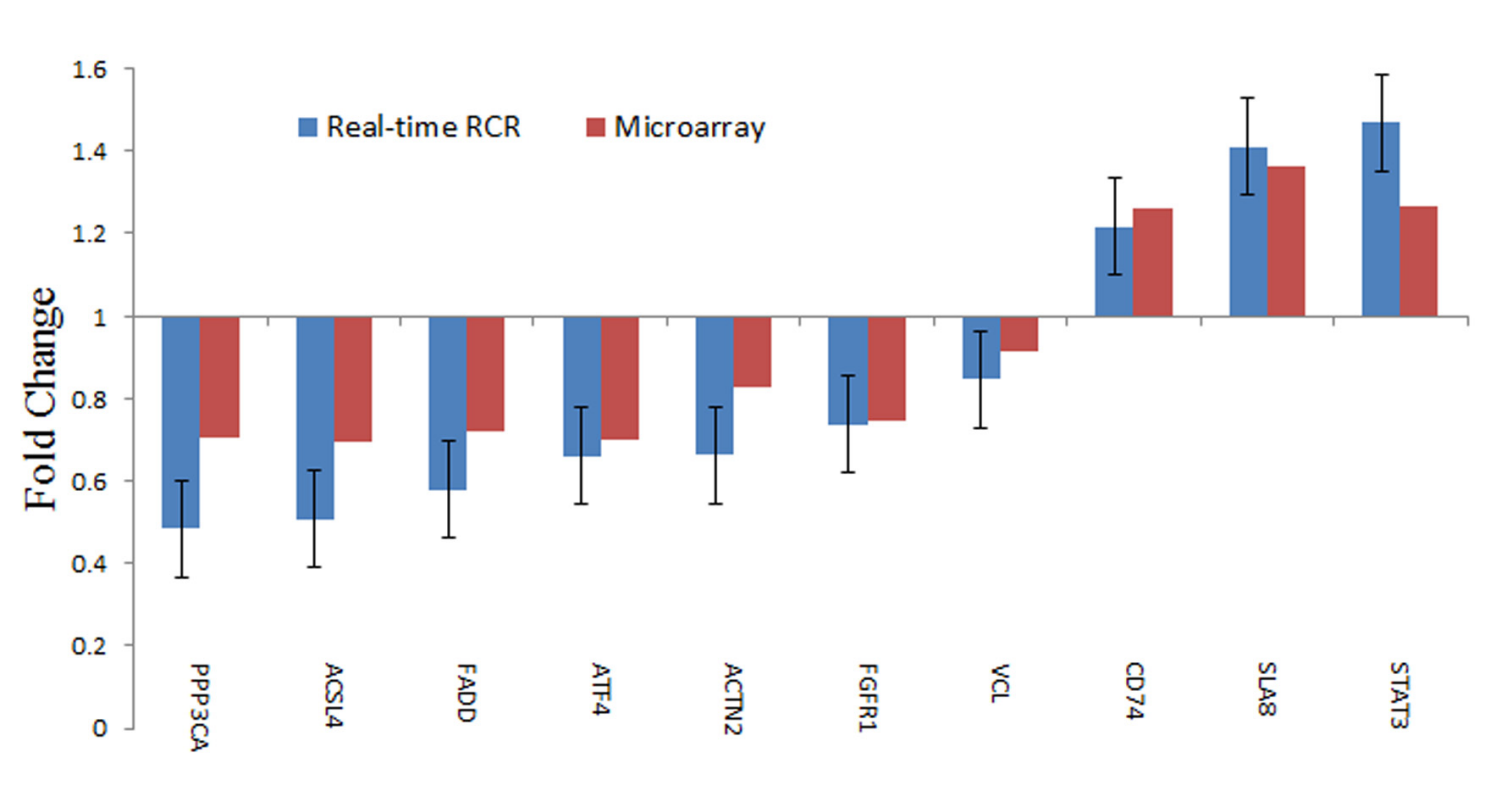
qRT-PCR for microarray validation. Ten genes that were either up- or down-regulated in the hearts of the HFHSD miniature pigs and were associated with metabolism, actin cytoskeleton signaling, and immune responses were selected for qRT-PCR. The data are shown as the fold differences in the HFHSD group versus the control group.

A swine whole-gene microarray were performed for comprehensive analysis of miniature pig heart mRNA expression profiles. Genes with ≥ 1.5-fold changes and P values of <0.05 were considered to be differentially expressed. Of the 23,935 genes represented on the chips, 1,022 were significantly differentially expressed, including 591 that were up-regulated and 431 that were down-regulated between the HFHSD and control groups. Unsupervised clustering analysis of the expression profiles showed highly consistent gene expression patterns for the two groups (Fig 3). Moreover, the gene expression patterns revealed by the microarray between the HFHSD and control pigs were validated with real-time QRCR to confirm the reliability of the microarray data (Fig 4). All the raw data were uploaded to Gene Expression Omnibus (GEO) with accession numbers GSE67890.

### The important proteins related to fatty heart and cardiac hypertrophy

**Fig 5:**
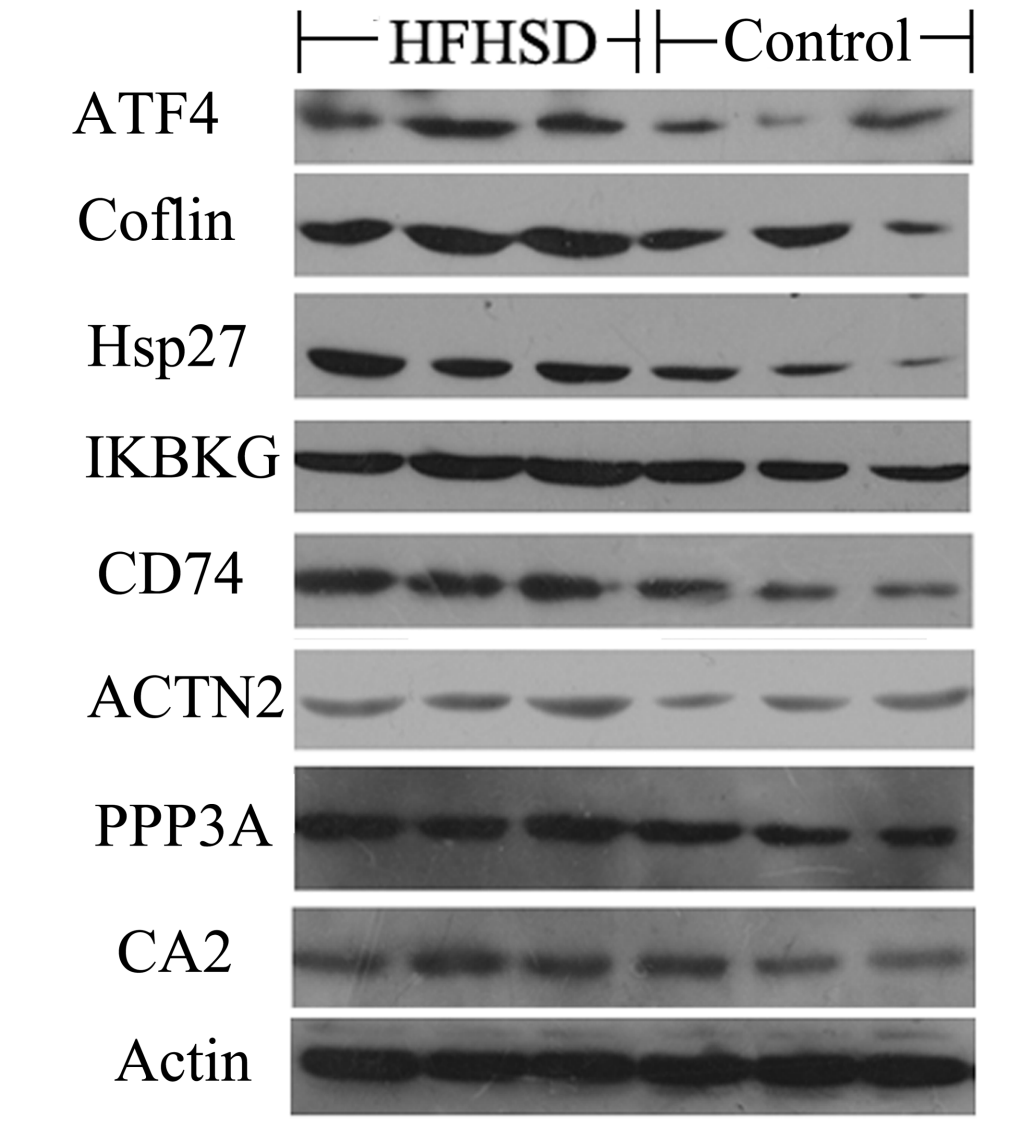
The important proteins related to fatty heart and cardiac hypertrophy in HFHSD group and control group.

The eight important proteins (ATF4, Coflin, Hsp27, IKBKG, CD74, PPP3A, ACTN2 and CA2) are selected from the differently expressed genes (P<0.05) between HFHSD group and control group. The cardiac myocyte hypertrophy pathway related gene and protein of ATF4 and ACTN2 are highly expressed in HFHSD group. In addition, inflammation pathway related HSP27 a heat shock protein, IKBKG a component of kappaB kinase inhibitor, and CD74 significantly up-regulated in the HFHSD miniature pigs in transcript and protein level. The eight proteins are highly expressed in HFHSD group which are closely related with fatty heart and cardiac hypertrophy (Fig 5).

To study the relationship between the genes and diet-induced heart disease, the mRNA transcripts were functionally classified by performing a search of the differentially expressed genes in the GO database. The functional annotations are presented in S1 Table. The differentially expressed GO terms (P<0.05) associated with the heart included the following: molecular functions, such as hydrolase activity, cytoskeletal protein binding, protein binding, lipase inhibitor activity, and glutathione peroxidase activity; and cellular components, such as the cytoplasm and chromosomes; biological processes, including humoral immune responses, neurotransmitter metabolic processes, regulation of microtubule-based processes, cell redox homeostasis, and the regulation of neurotransmitter levels (S1 Table).

### Analysis of pathways associated with differentially expressed genes in miniature pig hearts

To determine the relevant pathways, all 1,022 differentially expressed genes were evaluated with the SAS system (Shanghai Biotechnology Corporation, China), which includes the KEGG database. Many potential pathways were found to be affected in the HFHSD group (P<0.05). Signal transduction pathways were significantly activated, including the mitogen activated protein kinase (MAPK) pathway (P<0.01), the adipocytokine pathway (P<0.01), the chemokine pathway (P<0.05), the peroxisome proliferator-activated receptor (PPAR) pathway (P<0.05), and the TGF-beta signaling pathway. In addition, pathways related to cardiac hypertrophy were also significantly activated, such as focal adhesion (P<0.01), the apoptosis pathway (P<0.01), the axon guidance pathway (P<0.01), neurotrophin signaling pathways (P<0.01), actin cytoskeleton regulation (P<0.01), hypertrophic cardiomyopathy (P<0.05), calcium signaling (P<0.05), and cardiac muscle contraction (P<0.05). Pathways related to fatty heart were also significantly induced, including lysosomes (P<0.01), peroxisomes (P<0.01), pyruvate metabolism (P<0.01), glycerolipid metabolism (P<0.01), and arachidonic acid metabolism (P<0.05). Inflammatory signaling pathways were also promoted, including antigen processing and presentation (P<0.01), the B cell pathway and others (S2 Table). These pathways played important roles in fatty hypertrophy in the long-term HFHSD group miniature pigs.

## Discussion

### Long-term high-energy diets cause obesity and fatty hypertrophy in Bama miniature pigs

We have reported the inflammatory cell infiltration of nonalcoholic steatohepatitis in bama minipigs induced by a 23-month, high-fat, high-sucrose diet in miniature pigs [14]. In this study, we report the cardiac hypertrophy and fatty heart in miniature Bama pigs. Obesity is an important risk factor for fatty hypertrophy of the heart, and it exacerbates the detrimental effects of high-fat, high-energy diets on the heart, hastening myocardial fibrosis, apoptosis, and heart failure. After consuming a high-energy diet for 23 months, the HFHSD miniature pigs exhibited heart hypertrophy and cardiac myofibrillar swelling or atrophy. The cardiac hypertrophy in these animals increased cardiac pump function, decreasing the tension of the ventricular wall, thereby predisposing these animals to heart failure and sudden death, among other adverse effects [15]. Over-nutrition contributes to increased levels of plasma fatty acids and the ectopic accumulation of lipids in the myocardium, which results from increased fatty acid oxidation and the decreased efficiency of the obese heart [16]. In this study, the HFHSD group exhibited large amounts of fat deposits, cardiac myofibrillar disarray, and small amounts of inflammatory cell infiltration. In conjunction with insulin resistance, a relative lack of insulin and an increase in free fatty acids [17], abnormalities in glucose and lipid metabolism, and blood depression all play roles in damaging cardiac structures and functions [18]. Fatty acid accumulation, lipotoxic species and ROS cause dysfunctional myocardial contraction and even apoptosis; in addition, abnormalities in cardiac collagen can damage heart structures and function[19]. In the present study, the HFHSD miniature pig hearts showed slight increases in collagen and thicker blood vessel basement membranes. These minipigs are hyperinsulinemia and insulin resistance, which promote the synthesis of triglycerides and the deposition of fat. A lot of fat are stored as subcutaneous fat, visceral fat, and ectopic fat of important organ such as heart, liver. The effects of ectopic fat deposited in heart and liver are similar, causing disorganized structure, inflammation, mild fibrosis. At this stage, the injury of heart is mild, caused by long-term high energy diet, so we believe this model is good research material for the early stage cardiopathy. Furthermore, transcriptomic analyses identified the important genes and the pathways, which were annotated in online SAS database (Shanghai Biotechnology Corporation, China), related to fatty hypertrophy in the miniature pig hearts (S3 Table and S4 Table).

### Fatty hypertrophic heart-related signal transduction pathways in HFHSD miniature pigs

**Fig 6:**
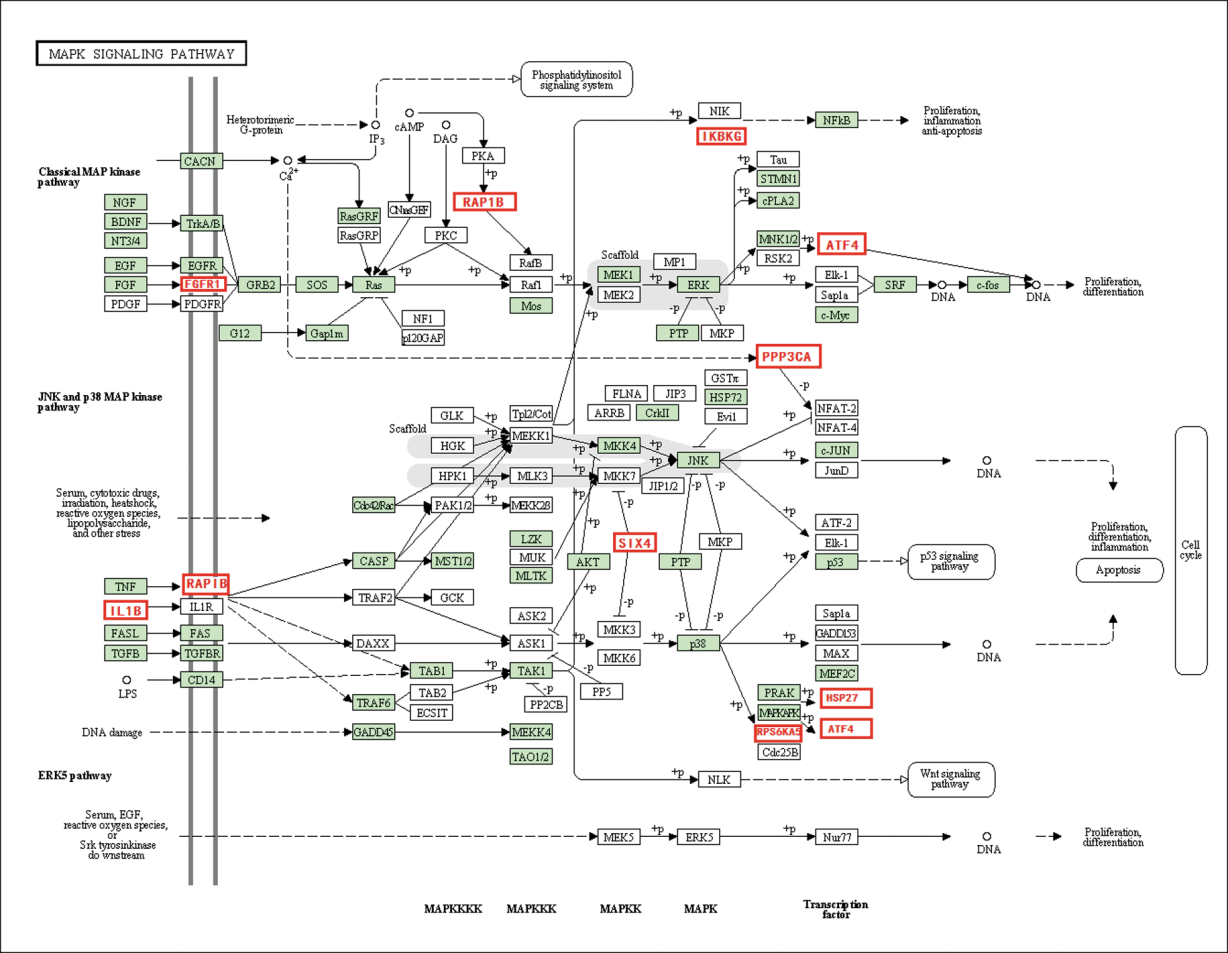
The genes related to the MAPK signaling pathway.

Transduction pathways play crucial roles in the development of fatty hypertrophic heart, participating in key steps in multiple pathways that link extracellular stimuli to intracellular signals [20]. In this study, the MAPK pathway, the adipocytokine pathway, the chemokine signaling pathway, the PPAR pathway (P<0.05), and the TGF-beta signaling pathway (P<0.05) were among the pathways that were significantly differentially regulated. In particular, the MAPK pathway directs and integrates a complex network that is involved in myocyte growth [21] and the activation of kinases, thereby controlling cell differentiation, growth, and apoptosis [22]. The MAPK pathway was significantly down-regulated in the HFHSD miniature pig hearts due to the long-term high-energy diet. Extracellular signals are transmitted into cells through the cell membrane receptors TNFRSF1A and FGFR1, which then activate SIX4 and RAP1B. SIX4 activates many fast-twitch muscle genes [22], and RAP1B, a small GTP-binding protein, regulates cellular processes, such as cell growth, adhesion, and differentiation [23]. Finally, several important genes downstream of the MAPK pathway were regulated in the studied miniature pigs, including IKBKG, ATF4, PPP3CA, and HSP27. IKBKG is a component of kappaB kinase inhibitor, which activates NF-kappaB and genes related survival and inflammation [24]. ATF4, a transcription factor, is widely expressed and is one of the DNA binding proteins that are involved in cell differentiation and proliferation [25]. PPP3CA encodes a multi-subunit phosphatase, and PPP3CA−/− mice exhibit changes in the brain consistent with hyperphosphorylation of the cytoskeletal protein substrate tau, along with memory impairment and susceptibility to immune suppression [26]. HSP27 is a heat shock protein, and it has important roles in cellular stress, atherosclerosis, oxidative stress, inflammation, and the production of cytokine and toll-like receptors [27]. The entire MAPK pathway was down-regulated in the HFHSD group miniature pigs, which may have been the result of heart hypertrophy (Fig 6).

### Differential genes of the actin cytoskeleton pathway

**Fig 7:**
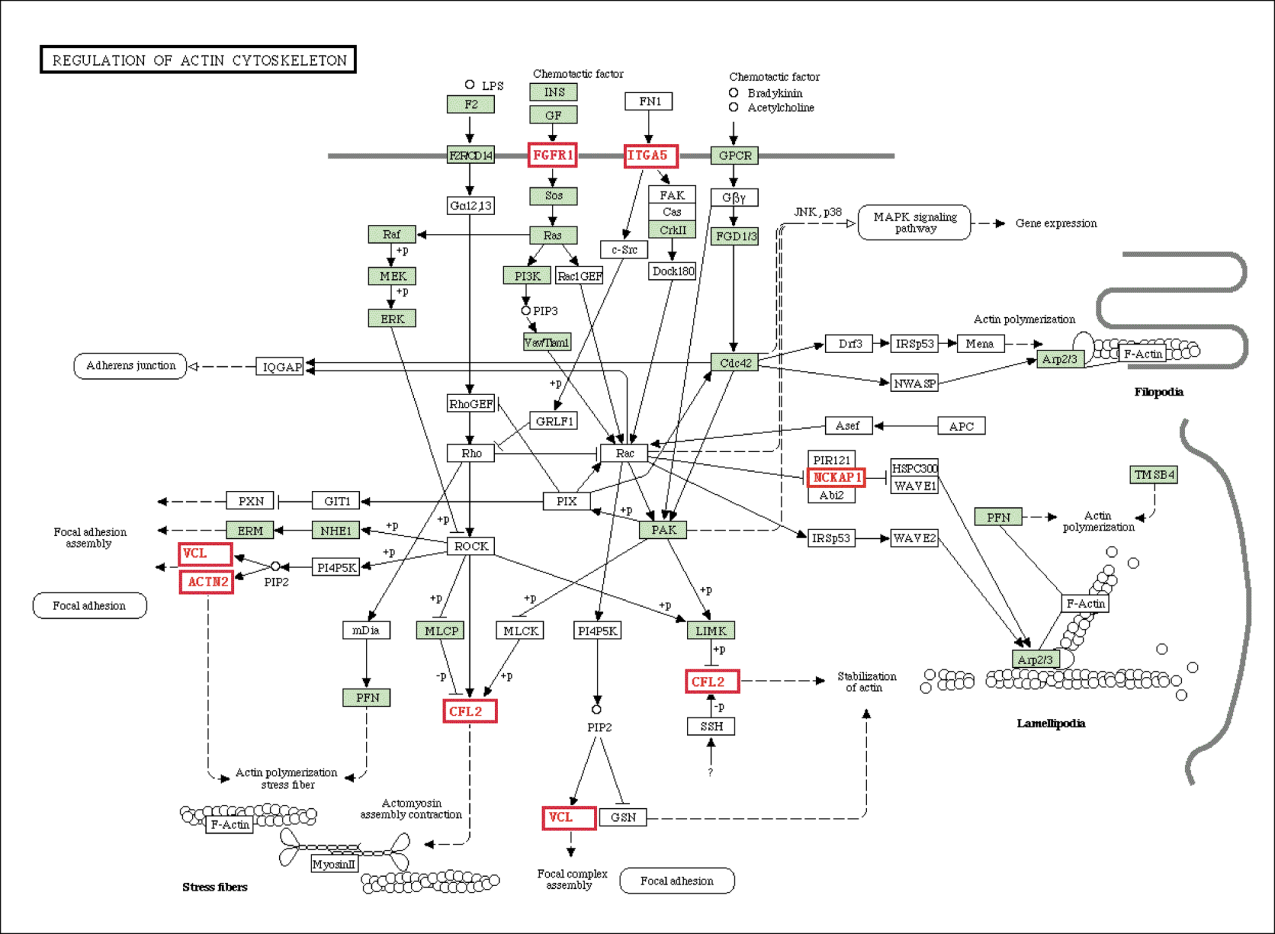
The genes related to the actin cytoskeleton pathway.

The cytoskeleton is essential to cardiac hypertrophy, not only because it maintains basic cell structure but also because it regulates various chemical signals and enzymes through signal transduction pathways. Compared with the control group, the actin cytoskeleton signaling pathways in the HFHSD miniature pig hearts were significantly differentially expressed. FGFR1, a member of the fibroblast growth factor receptor family, is associated with mitogenesis and differentiation [28], and ITGA5, an integrin receptor, is known to affect adhesion and participate in cell surface-mediated signaling [29]. These cell membrane proteins activate a cascade of downstream signals. Downstream genes, including ACTN2, VCL, MYL2 (LOC733637) and CFL2, directly influence cell size and structure. ACTN2 is a cytoskeletal protein that is localized to the Z-disc and analogous dense bodies in the heart, where it anchors actin filaments [30]. The vinculin (VCL) gene encodes a cytoskeletal protein that functions in cell and matrix junctions and anchors actin to the cell membrane [31]. The MYL2 gene encodes the cardiac myosin light chain protein, and previous studies have shown that a mutation in this gene results in left heart chamber hypertrophic cardiomyopathy [32]. CFL2 encodes a type of actin rod protein that is involved in regulating actin filaments [33]. The cytoskeleton receives extracellular signals and transmits them to the cell, thereby influencing focal adhesions, myocardial contractility, actin polymerization, and the microtubule network (Fig 7).

### Genes involved in the immune response

**Fig 8:**
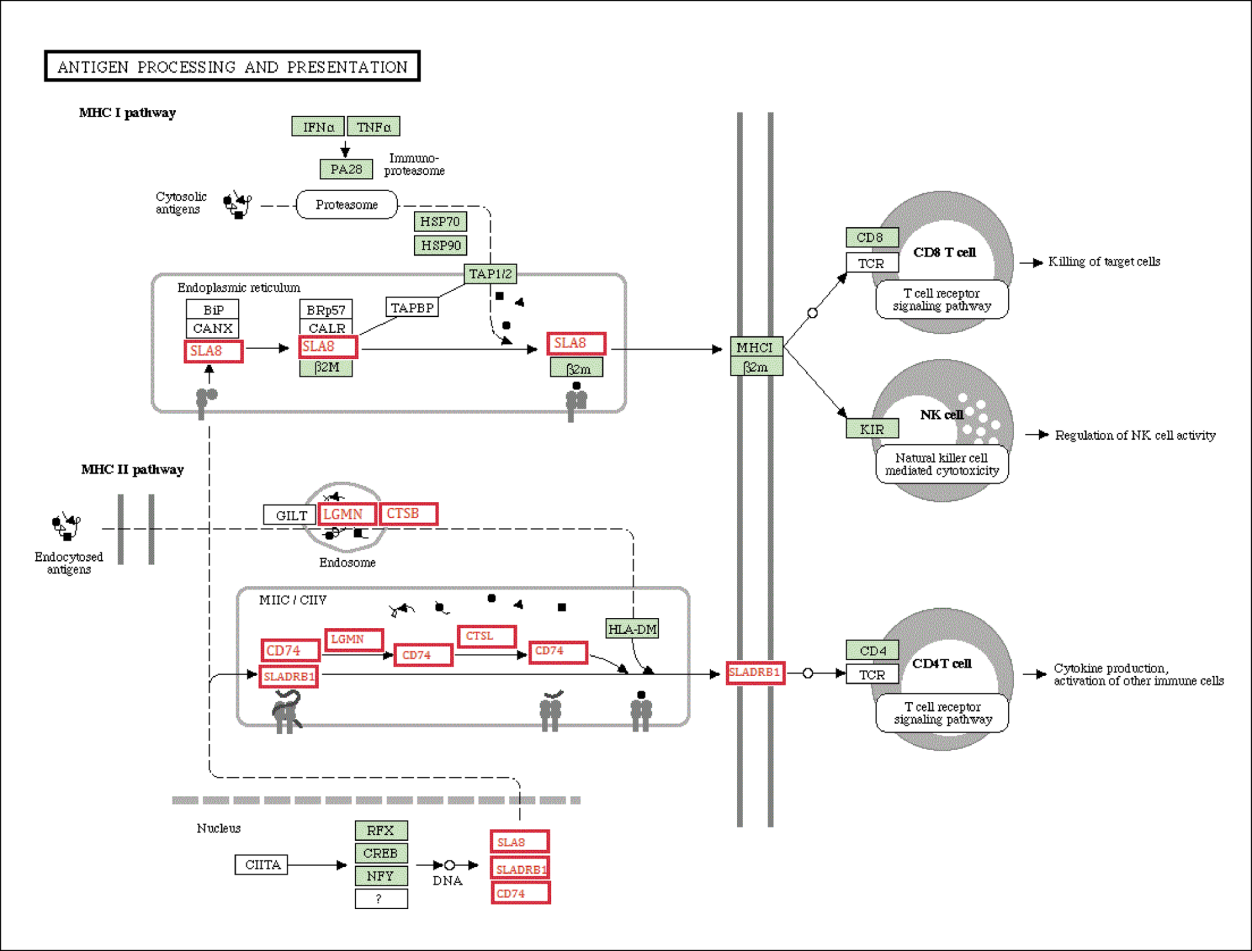
The genes related to the antigen processing and presentation pathway.

Inflammation in the heart is widely considered to be an important factor underlying mechanical pressure overload, heart disorders and metabolic syndrome [34], and it can cause detrimental effects on the cardiac extracellular matrix and drive macrophage invasion and heart fibrosis [35]. In this study, H&E staining revealed inflammatory cell invasion and slight fibrosis in the HFHSD miniature pig hearts. The antigen presentation-related genes SLA-8 [36], CD74 [37], and SLA-DBR1 [38], which are important for immune responses and inflammatory cytokine production, were significantly up-regulated in the HFHSD miniature pigs. Interestingly, the genes LGMN and CTSB, which play important roles in the endosomal processing of endocytosed antigens, were down-regulated. The LGMN gene encodes a cysteine protease that presents bacterial and endogenous peptides to major histocompatibility complex (MHC) class II molecules in the lysosomal and endosomal systems [39]. The CTSB protein is a lysosomal cysteine proteinase that is comprised of a dimer of disulfide-linked heavy and light chains that are made from the same protein precursor that is involved in proteolytic processing [40]. The immune system is activated in the HFHSD miniature pig heart, which leads to the release of inflammatory factors, increased cytokine production, immune cell activation and inflammation-related ROS production, resulting in the development cardiomyocyte disorders, hypertrophy, and other heart diseases (Fig 8).

## Conclusion

In summary, this study is the first report of fatty heart and cardiac hypertrophy induced by a long-term, high-fat, high-sucrose diet in Bama miniature pigs. The studied pigs exhibited obesity and hypertrophic fatty heart, accompanied by inflammatory cell invasion and mild fibrosis. Analyses of the Bama miniature pig heart transcriptome indicated that signal transduction, such as that associated with the MAPK, PPAR and cytokine pathways, was directed toward actin cytoskeletal signaling and inflammatory pathways. Furthermore, the expression of heart-related genes, including STAT3, ACSL4, ATF4, FADD, PPP3CA, CD74, SLA-8, VCL, ACTN2 and FGFR1, which may play important roles in the processes underlying fatty hypertrophy, may be induced by long-term high-energy diets.

## Supporting Information

S1 Table All GO analysis information for the differentially expressed genes. (XLS)

S2 Table All pathway information for the differentially expressed genes. (XLS)

S3 Table Selected genes related to fatty heart and cardiac hypertrophy induced by the high-energy diet. (XLS)

S4 Table All genes detected in the HFHSD and control groups. (XLS)
